# The Punatsangchhu-I dam landslide illuminated by InSAR multitemporal analyses

**DOI:** 10.1038/s41598-020-65192-w

**Published:** 2020-05-19

**Authors:** Benedetta Dini, Andrea Manconi, Simon Loew, Jamyang Chophel

**Affiliations:** 1 Department of Earth Sciences, ETH Zurich, Switzerland; 2 PHPA-I, Wangduephodrang, Bhutan

**Keywords:** Natural hazards, Environmental sciences

## Abstract

We use multitemporal analyses based on Synthetic Aperture Radar differential interferometry (DInSAR) to study the slope adjacent to the large Punatsangchhu-I hydropower plant, a concrete gravity dam under construction in Bhutan since 2009. Several slope failures affected the site since 2013, probably as a consequence of toe undercutting of a previously unrecognised active landslide. Our results indicate that downslope displacement, likely related to the natural instability, was already visible in 2007 on various sectors of the entire valley flank. Moreover, the area with active displacements impinging on the dam site has continuously increased in size since 2007 and into 2018, even though stabilization measures have been implemented since 2013. Stabilisation measures currently only focus on a small portion of the slope, however, the unstable area is larger than previously evaluated. Highly damaged rock is present across many areas of the entire valley flank, indicating that the volumes involved may be orders of magnitude higher than the area on which stabilisation efforts have been concentrated after the 2013 failure. The results highlight that satellite-based DInSAR could be systematically used to support decision making processes in the different phases of a complex hydropower project, from the feasibility study, to the dam site selection and construction phase.

## Introduction

Narrow valleys of deeply incised mountainous ranges represent an ideal set-up for the construction of dams, because they allow the creation of large reservoirs with the minimum structure width. Occasionally, valley constrictions caused by landslides provide a deep, narrow gorge bounded by steep sides, and offer, at least at first sight, the picture of an appealing site for dam construction^1,2^.

However, gravitational movements at the abutment of a dam can also have critical consequences. A notable case of a dam built directly on a landslide is the Beauregard dam, in northwest Italy. Here a deep-seated gravitational slope deformation impinges directly on a 132 m high concrete arch-gravity dam^3^. The landslide, with the basal shear zone at a depth of 240 m in the lower parts^3^ and a volume of 200 million m^3^ ^4^, had its onset about 10 ka ago and it experienced reactivation after the filling of the reservoir in 1958, showing significant displacements across a large portion of the slope. Though this dam is still operational, the recognised necessity of maintaining safety and of avoiding a potential catastrophic failure, has led to a substantial decrease of the dam maximum operating capacity. With a planned reservoir capacity of 70 hm^3^ and a height of 1765 m a.s.l., the dam has been kept in operation at a capacity limited to 2.3 hm^3^ and a reservoir height 62.5 m lower than originally intended.

Landslides and dams interact also when the dam is built in the vicinity of pre-existing instabilities. The modifications of the hydrological conditions at the toe of a slope caused by induced river flow changes and by the presence of a reservoir are associated with changes in the distribution of pore water pressure within the slope, leading to new water table conditions and fluctuations that get superimposed on natural seasonal cycles^3,5,6^. The consequences of catastrophic slope failures resulting in deposition of material into a dammed lake can be extremely serious and have impacts a long way downstream of the site. Though the list of landslides built in close proximity to dams is long^1^, an extraordinary case is the Vajont dam, in northeast Italy, the story of which is tragically known for its devastating epilogue. In October 1963, a 250 million m^3^ rock mass slid into the Vajont reservoir, causing a wave that destroyed an entire village and killed around 2000 people. It has been accepted in the literature that the failure was caused by the reactivation of a prehistoric rock slide^7^, the identification of which was not disclosed nor fully investigated at the design stage^8^. In the first half of the past century, in a rapid growth of the hydropower sector, a large number of dams were built around the world without adequate investigations and without a full understanding of either the geological and geotechnical problems or of the potential catastrophic consequences that negligence in addressing them could cause^1^. However, nowadays, little space for disagreement is found in the literature regarding the utmost importance of carrying out appropriate investigations leading up to the choice of a dam site. The monitoring of slopes impinging on or in the vicinity of a planned or existing hydropower plant is an important aspect in the identification of potential instabilities.

Developing countries characterised by mountainous terrain and high relief, such as many Himalayan countries, are experiencing an increased push towards the expansion of hydropower, which is seen as a promising revenue source, thanks to the exploitation of a seemingly favourable geographical setting. In recent years, a large initiative launched by the governments of Bhutan and India, has led to the planning of several, large hydropower plants to be built in Bhutan by 2020, with an expected overall power output of 10000 MW^9^. 80% of the energy produced will be sold to India^10,11^, the revenue coming from this sector already amounting to 25% of Bhutan GDP^12,13^ and expected to soar in the future. India largely controls the planning and construction of hydropower plants, being the main consumer of most of the electricity produced, and grants loans to Bhutan to cover for project costs. Thus, the political aspect of this rapid hydropower growth has a profound effect on the assessment and management of environmental hazards. Recent studies have shown that risk management and disaster preparedness have been sidelined in hydropower planning processes across the Himalayas^14^. This may be due not only to failure in producing adequate geological and geotechnical assessments, but also to the fact that “a blind-eye to environmental risks facilitates the appropriation of economic benefits by powerful interest groups”^14^. In the same study, the author highlights the importance of encouraging an increase in knowledge about risks in order to create a basis for a more aware and informed debate around hazardous hydropower projects. Bhutan, despite its recent hydropower history, is not free from related disasters. In August 2015, for example, a landslide occurred at the Mangdechhu site burying 5 workers^15^.

Another potentially hazardous project is the Punatsangchhu-I. The 1’200 MW Punatsangchhu-I hydropower project, located 20 km south of Punakha and 50 km east of the capital Thimphu, is part of the 10’000 MW initiative and it comprises a 134 m high gravity concrete dam^16^, two diversion tunnels and a 10 km long headrace tunnel. The project began in November 2008 and it was initially scheduled to be completed by 2015. Lacking geological and geotechnical investigations have, however, caused large delays and a large increase in the overall costs, with the project not yet being completed at time of writing. A large failure occurred in July 2013 on the east-facing slope, causing the construction works to come to a halt in favour of a huge effort aimed at stabilising the lower portion of the slope. More recently, in January 2019, another failure injured a worker and caused additional damage^17^. An incomplete understanding of the nature and extent of the real problem, during planning, design, excavation and construction phases has led to costly delays and the potential future amplification of an existing natural hazard.

In the past two decades, synthetic aperture radar differential interferometry (DInSAR) has gained increasing importance in imaging unstable slopes, due to its suitability for retrieving information over large and inaccessible areas, whilst potentially ensuring a continuous, versus point-like, overview of the displacements affecting the illuminated ground surface e.g^18–22^. In the specific case of the interaction between dams and landslides, the usage and usefulness of this technique has been demonstrated for example for the Beauregard landslide (Ground Based DInSAR^3^,) and for various landslides in China, such as the Guobu landslide upstream of the Laxiwa power station^23^, the Badong county landslides^5^ and the Xintan landslide^24^ upstream of the Three Gorges dam. These studies show that DInSAR is able to show significant displacements over sectors of the monitored instability for which no prior knowledge existed and that the widespread information of displacements can facilitate interpretations of the landslide’s behaviour and mechanisms. Moreover, DInSAR allows the monitoring of spatial and temporal evolution of the displacements and to make correlations between water level changes and seasonal landslide movements, this enabling the understanding of the impact of water level changes on the reactivation of pre-existing landslides.

**Figure 1:**
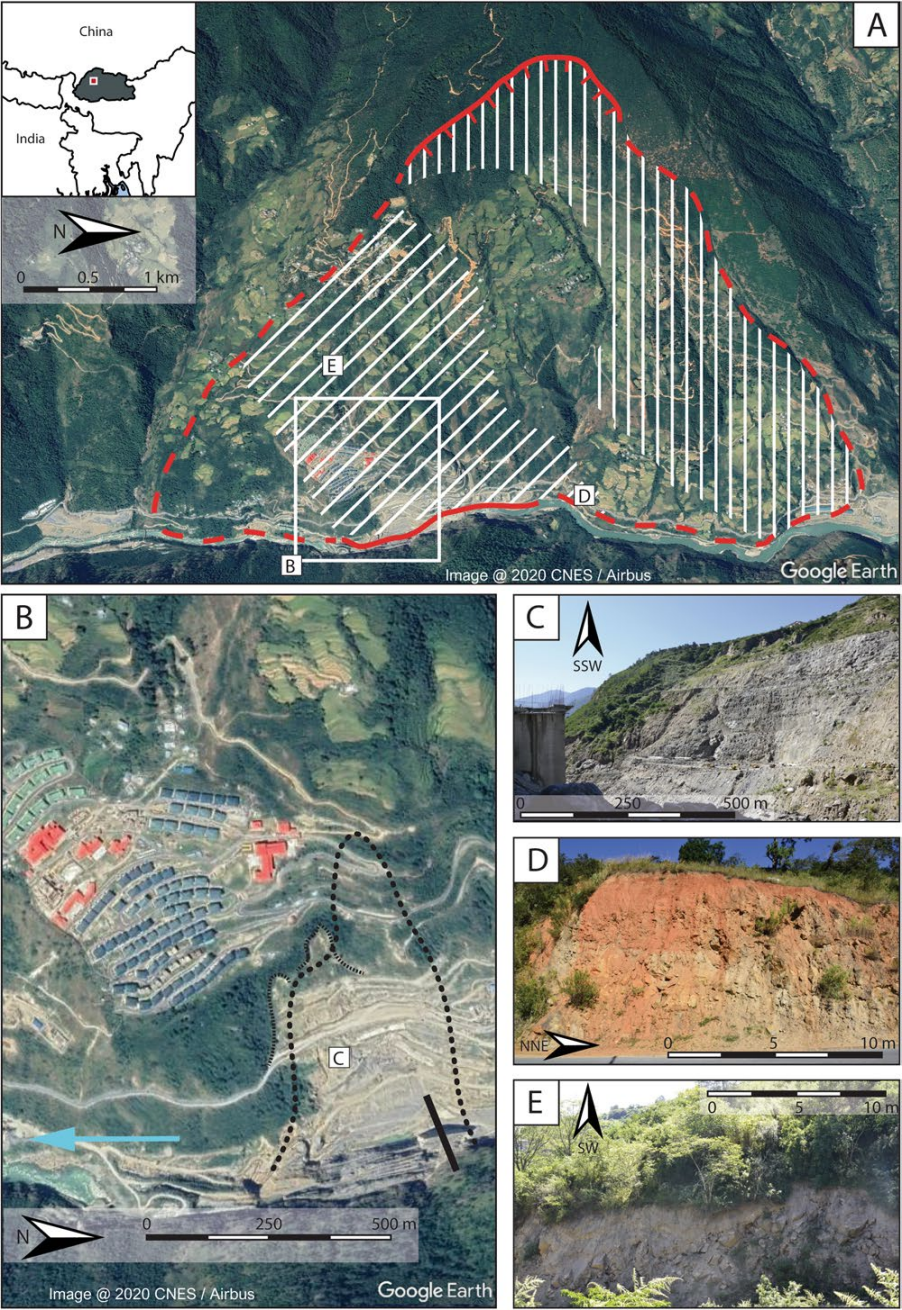
(**A**) Google Earth CNES/Airbus image of the area of study. Red lines with ticks represent the head scarp, presumed (red dashed) and known (red continuous) boundaries. Hatched area represents damaged rock (diagonal) and undisturbed residual soil (vertical). White square is the area in B. Inset shows the regional setting. (**B**) Google Earth CNES/Airbus image of white square in A. Black line represents the dam axis, thick dotted line indicates the 2013 failure area, thin dotted lines indicate scarps likely related to the 2013 failure. (**C**) Photo of the 2013 failure area. Given the angle from which the photo is taken, only the southern boundary is well visible, marked by scarps and the appearance of vegetation on stable ground. On the left of the photo, the construction of the left dam abutment is visible. (**D**,**E**) intensely damaged rock, location shown in A.

In this study, we measure the spatial and temporal evolution of the surface displacements over an area of 15 km^2^ at the Punatsangchhu-I dam site (Fig. 1) before and during the construction works, with 11 years of satellite SAR data acquired from 2007 to 2018 (with a three-and-a-half year gap). A multi-temporal DInSAR analysis highlights that the valley flank was already affected by displacements before the start of the construction of the dam and shows an increase of the surface displacement rates subsequent to the beginning of the works. The spatial coverage of the measurements also highlights that the instability is not only affecting the area immediately around a large failure which occurred in 2013, but it covers a much larger area of about 8 km^2^ in total. This could have critical implications for the volumes of rock involved, which could be of the order of hundreds of millions of m^3^ in the worst-case scenario. Our measurements and analyses underline that remote sensing techniques, and in particular DInSAR, an established method for detecting ground displacements with millimetric accuracy^21^, could have allowed stakeholders to identify a potential problem at this dam site and to understand its extent before the instalment of such critical infrastructure. The increased availability of remote sensing data has now the potential to play an important role in filling a gap in the site-specific knowledge caused by insufficient or inadequate investigations and can significantly increase the transparency and public awareness around such large projects.

## Results

**Figure 2:**
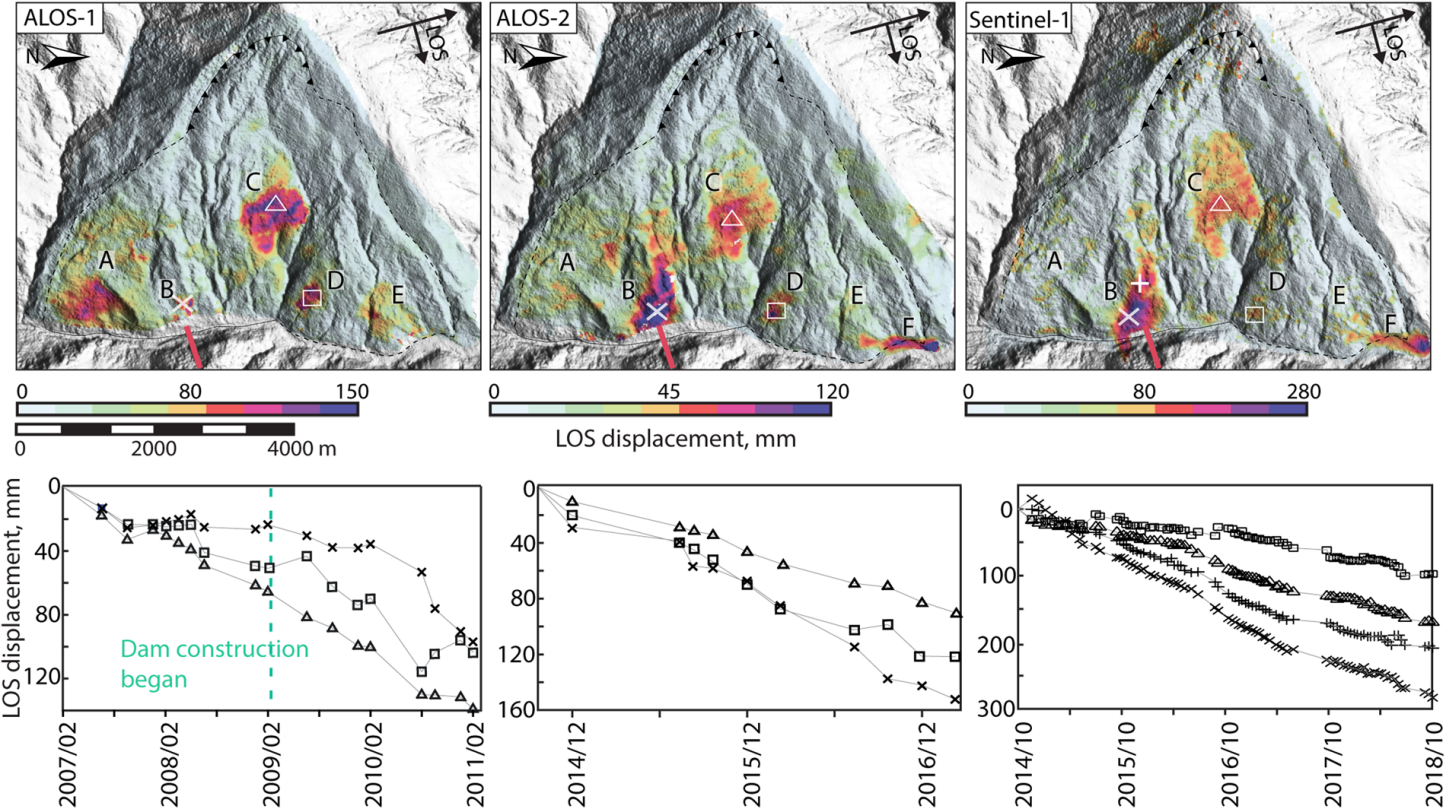
The top three panels show LOS cumulative displacements in the three sensors. Colour scales are different and non-symmetrical to highlight areas of highest displacements, with high variability in different sectors and in the three sensors. Letters indicate the sectors described in section 2. Left and centre panels at the bottom show the time series of ALOS-1 and ALOS-2 of three points in sectors B, C, and D. Sentinel-1 panel at the bottom right shows the time series for the same points as in the other two sensors, with the addition of one point in sector B (cross). Points shown in the corresponding panels above. Dam axis in red. ALOS-1 and ALOS-2 SAR imagery obtained from JAXA (Dataset ©JAXA/METI ALOS PALSAR L1.0 2007, 2008, 2009, 2010, 2011, 2014, 2015, 2016, accessed through https://auig2.jaxa.jp/openam/UI/Login, 2016 and 2017). Copernicus Sentinel data [2014, 2015, 2016, 2017, 2018] accessed through the ESA Copernicus open access hub https://scihub.copernicus.eu/dhus/#/home, 2017, 2018. InSAR processing done with the software SARScape from Sarmap.

The observation period spans from February 2007 to October 2018 (Table 1), with ALOS-1 covering a four-year period between February 2007 and February 2011, ALOS-2 a two and a half years period between September 2014 and February 2017 and Sentinel-1 a four years period between October 2014 and October 2018. In the following, we show how the InSAR signal highlights different sectors of the entire valley flank that are affected by cumulative displacements throughout the observation period (Fig. 2). Such sectors are well detected because they are characterised by spatially continuous patterns of downslope displacements (generally >20 mm). Each of these sectors increase in areal extent and show cumulative displacements during the periods illuminated by the three sensors and some of them also show displacement rate changes in the time series.

**Table 1:** Data used.

Satellite	Band	Incidence angle	Track/ frame	Orbit type	Images number	Period
ALOS-1	L	~38.75°	502/530	A	18	01 Feb 2007- 12 Feb 2011
ALOS-2	L	~31.39°	154/540	A	11	19 Sep 2014 - 2 Mar 2017
Sentinel-1	C	~39°	114	A	79	25 Oct 2014 – 4 Oct 2018

### Areas, cumulative displacements and average velocities for each sensor’s period

Sector A, as seen by ALOS-1, is characterised by an area of downslope cumulative displacements in the period February 2007 – February 2011 of >10 cm (with average velocity of roughly 3 cm/year estimated over the whole period) covering about 0.5 km^2^. A larger area of 2.3 km^2^, encompassing the zone of greater displacements and extending 1.2 km upslope from its upper margin, shows cumulative displacements between 5 and 10 cm (with average velocity of roughly 2.5–3.5 cm/year estimated over the whole period). This larger extent is well matched by the observations retrieved with ALOS-2, albeit cumulative displacements between September 2014 and February 2017 are on the whole lower than those shown by ALOS-1. The extent of the displacements in this sector becomes patchier in Sentinel-1 observations, between October 2014 and October 2018, and the boundaries less easily traced, with greatest displacements restricted to the toe of the slope and at around 1360 m a.s.l.

Sector B (immediately upslope of the dam site) in ALOS-1 shows two different areas with displacements largely above 5 cm (with average velocities around 1.5 cm/year), each roughly covering 0.1 km^2^. The upper one presents lower cumulative displacements with maxima around 7 cm (only faintly visible in Fig. 2 due to the scale, about 50 m upslope of point x, with average velocities around 2 cm/year). The lower one, at the toe of the slope, is characterised by larger cumulative displacements (visible in Fig. 2 around point x), reaching 15 cm by February 2011 (and average velocities exceeding 2.5 cm/year). ALOS-2 shows a fivefold areal increase by February 2017, the overall extent reaching 1 km^2^, comprising the two areas observed with ALOS-1. Displacements are largely above 8 cm and reach up to 22 cm in the lowest half (with average velocities largely around 2.5 cm/year and up to 8 cm/year in the lowest half). Although the boundaries are more jagged in the upper parts in Sentinel-1 data, the area is comparable to that seen in ALOS-2 and displacements exceed 30 cm in the lower half by October 2018 and similar average velocities to ALOS-2.

ALOS-1 displacements over the whole observation period in sector C cover an area of 1.2 km^2^. Displacements are largely above 5 cm and exceed 15 cm in the central parts (with average velocity around 3 cm/year). This area increases to almost 2 km^2^ in ALOS-2 displacement map, with values largely above 3 cm and exceeding 9 cm in places (reaching average velocities of 3–4 cm/year) and a shift towards the south. The area observed with Sentinel-1 is similar in extent (1.9 km^2^), though again it presents a shift, this time upslope and towards the north. Displacements are largely above 5 cm and reach up to 17 cm in the centre (with average velocities up to 4.5 cm/year).

Sector D covers an area of 0.4 km^2^ in ALOS-1 cumulative displacement map. Displacements are on the whole above 5 cm and increase up to 13 cm towards the lower half of the area (with average velocities of 3–3.5 cm/year). The area is comparable in size in the ALOS-2 displacement map, with displacements above 3 cm and up to 14 cm (and average velocities of up to 5–6 cm/year). A small increase in extent (0.7 km^2^) and a patchy appearance of the displacements is seen in Sentinel-1, with displacements on the whole above 5 cm but up to 11 cm (with average velocities up to 2–3 cm/year).

Sector E as illuminated by ALOS-1 is characterised by an area of 0.5 km^2^. Cumulative displacements between February 2007 and February 2011 lie between 7 and 10 cm (with average velocities up to 2.5 cm/year). ALOS-2 suggests that the area shrinks in its period of observation to 0.3 km^2^, with cumulative displacements above 3 cm in general and up to 7 cm at elevations between 1250 and 1280 m a.s.l. (with average velocities generally around 2.5 cm/year). Sentinel-1 displacements over this sector appear patchy, with unclear boundaries. Cumulative displacements between 10 and 12 cm represent the maxima and are found again between 1250 and 1280 m a.s.l. (with average velocities between 2 and 2.5 cm/year).

Sector F is not visible in the cumulative displacement map of ALOS-1, but it appears in the period of observation of the other two sensors, September 2014 to October 2018. With ALOS-2 the area is seen as covering an extent of 0.16 km^2^, with cumulative displacements up to 16 cm (with average velocities up to 7–8 cm/year). The area increases to 2.2 km^2^ affected by cumulative displacements of up to 32 cm, as observed with Sentinel-1 (with average velocities around 7 cm/year).

### Time series

Time series are analysed and described here for sectors B, C and D, and are shown in the lower panels in Fig. 2.

**Figure 3:**
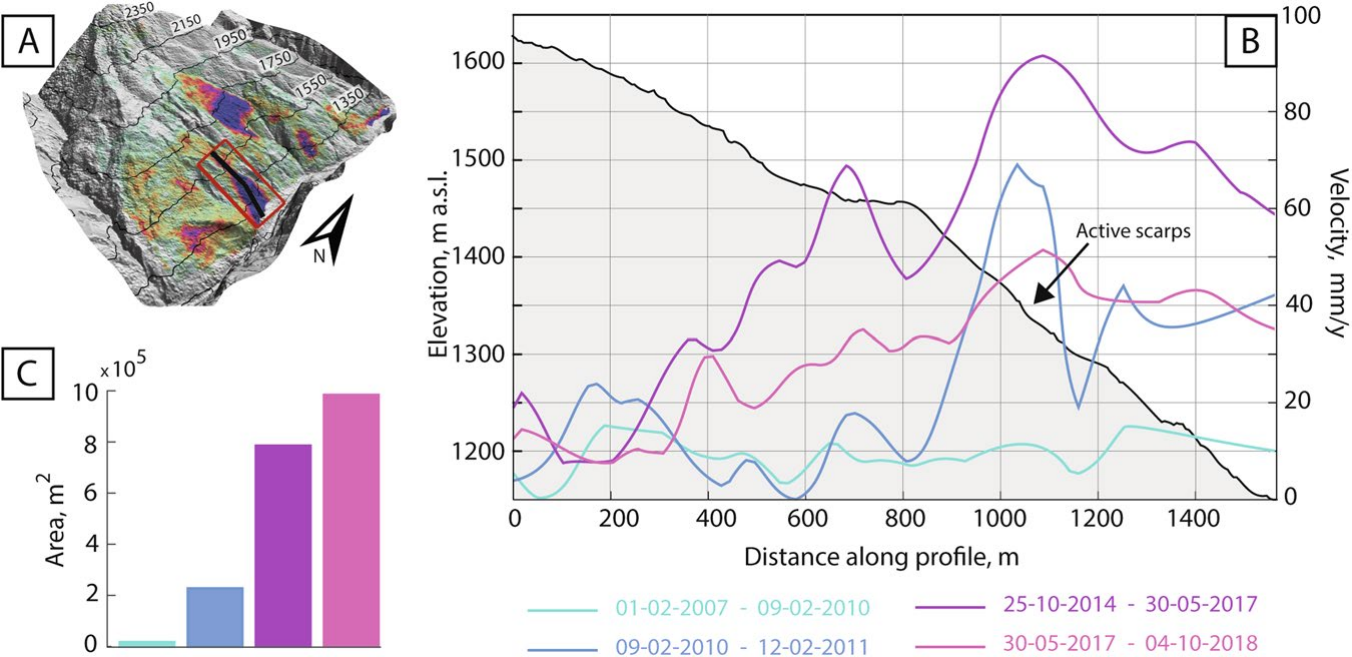
(**A**) 3D map inset shows the sum of cumulative displacements (stack) of all sensors over the entire slope, in red polygon the area of sector B and in black the profile are shown. (**B**) Velocities along the profile intersecting sector B. Slope profile shown in black. The different colours represent four periods over which velocities are calculated. (**C**) Histogram of the active displacements area size. (ALOS-1 and ALOS-2 SAR imagery obtained from JAXA (Dataset ©JAXA/METI ALOS PALSAR L1.0 2007, 2008, 2009, 2010, 2011, 2014, 2015, 2016, accessed through https://auig2.jaxa.jp/openam/UI/Login, 2016 and 2017). Copernicus Sentinel data [2014, 2015, 2016, 2017, 2018] accessed through the ESA Copernicus open access hub https://scihub.copernicus.eu/dhus/#/home, 2017, 2018. InSAR processing done with the software SARScape from Sarmap.).

In sector B, the ALOS-1 time series of a point at roughly 1320 m elevation (x in Fig. 2) shows a non-linear trend. In the first half of the period, from February 2007 to February 2009, the trend is on the whole shallow (though higher displacement rates characterise the period up to October 2007), followed by two acceleration phases during the second part of the period, from February 2009 to February 2010 and then more markedly from February 2010 until February 2011. The trend for the same point as seen with ALOS-2 and Sentinel-1 shows a roughly twofold increase in velocity in comparison to the period February 2009-February 2011 (see section 2.3 and Fig. 3) and linearity between September 2014 and June 2017. Sentinel-1 shows then a decrease in the displacement velocity after June 2017, though linearity is again seen through this last period. Velocities in this sector and their variations in time are described in detail in section 3.3.

On the whole, the time series in the centre of sector C show linear trends. The cumulative displacements observed over the different periods of observation of the three sensors show that the velocity remained similar throughout the whole period, lying relatively steady between 32 mm/year (ALOS-1) and 37 mm/year and 45 mm/year (ALOS-2 and Sentinel respectively). Some small deviation from a linear trend may be observed ALOS-2 images and in Sentinel-1 time series.

The time series of a chosen point in sector D generated with ALOS-1 images is more strongly non-linear. An initial phase with a lower velocity is visible up to end of June 2009. After that, a period of acceleration is observed until August 2010. We observe a change in average velocity from 20 mm/year, to 70 mm/year for ALOS-1 in the periods identified, and to around 50 mm/year as observed with ALOS-2 and then decreasing again to 30 mm/year through the rest of the period.

### Velocities along a profile in sector B

Velocities were calculated along a profile crossing sector B, in the immediate proximity of the dam site, from west to east and from 1620 to 1150 m a.s.l., using ALOS-1 and Sentinel-1 data, given the partial overlap between ALOS-2 and Sentinel-1. Figure 3 shows the velocity calculated assuming linear displacement rates over four different periods, the latter defined by changes in the observed trends (as seen in the lower panels in Fig. 2). Between February 2007 and February 2009, the velocity remained relatively constant along the profile, at around 20 mm/year or less. From February 2009 the velocity increased remarkably between 1450 and 1350 m a.s.l. from less than 20 mm/year to over 60 mm/year, and below 1250 m a.s.l. it increased to over 30 mm/year. A further acceleration is observed from October 2014 to May 2017. During this period, we observe that between 1600 and 1450 m a.s.l. the velocity shows a downslope increase, reaching 70 mm/ year. Moreover, after a 100 m wide, flat section at 1450 m a.s.l., the velocity increases downslope up to about 90 mm/year at 1350 m a.s.l. Although downslope of 1300 m elevation the velocities appear lower than in the upslope sections during this period, we still observe an overall increase from the previous period. Only in the last period, from May 2017 to October 2018 we observe a decrease in velocities: even though there are still parts of the slope with increasing downslope gradients, the overall velocities along the profile are lower in comparison to the previous interval, but higher in comparison to the beginning of the observations.

### Geomorphological observations

The study area of this work, 20 km south of Punakha, is located geologically within the rocks of the Greater Himalayan Zone^25^, comprising metasedimentary rocks including quartzite, muscovite-biotite-garnet schist and paragneiss^26^. The mean slope angle of the entire valley flank is 23°, with sectors A to F having mean slope angles of 22°, 26°, 23°, 21°, 21° and 16° respectively.

Analysis of the high-resolution DSM reveals the presence of an old head scarp, with heights varying between 30 and 100 m. The scarp can be followed for an arched path of 3 km, from 2030 m a.s.l. in the south, to 2430 m a.s.l. at its highest point below the ridge top, to 2080 m a.s.l in the north. A convex profile is also present below 1600 m a.s.l., with bulging in the lower parts of the slope. Erosion at the toe, visible in a Google Earth image prior to 2009, indicates active river incision, particularly evident in the south; moreover, in the northern half of the slope a marked curvature in the river bed towards the opposite side of the valley is also present. An abundance of deeply incised channels, visible both in the DSM and in the optical images, suggests high erodibility of the material. This dense network of channels is coupled with high roughness of the topography, which confers an irregular morphology to the slope.

During a field visit in October 2017, we observed intensely damaged rock across a large part of the slope (Fig. 1). This extends from the area of the village, upslope to the smaller settlement on the southern flank of the slope at around 1800 m a.s.l. and then north into the middle of the slope. Outcrops along the farm road in the northern half of the slope show relatively undisturbed residual soils, with preserved parent rock structure (vertical hatches in Fig. 1A). Fresh scarps related to the 2013 slope reactivation are observed below the village at elevations going from 1250 m a.s.l. in the south, intersecting the road, to 1340 m a.s.l.at the highest point, to 1290 m a.s.l. in the north (Fig. 1B). These scarps, with an estimated height between 10 and 20 m, are also visible in Google Earth images (shown as thin dotted lines in Fig. 1B). A large deposition is also observed below the village, this possibly corresponding to the accumulation of the 2013 event (Fig. 1C). The lower parts of such deposits are intensely reworked by activity at the site.

### *In-situ* data as validation for InSAR measurements

**Figure 4:**
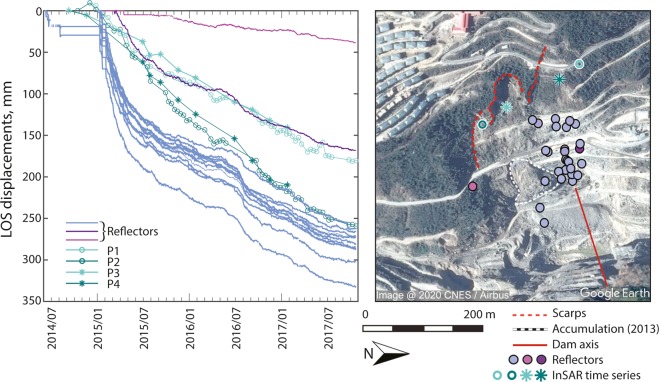
Left: comparison between *in-situ* data (solid lines) and satellite-based time series (lines with markers). Right: area of 2013 and 2016 failure with location of total station reflectors and sampling points of satellite observations (Background Google Earth CNES/Airbus imagery).

A total station installed at the site on the left abutment on the dam has recorded the movements of reflectors placed within the 2013 failure area. 39 reflectors were installed between the deposit of the 2013 failure and the scarps below the village, these covering an area 300 m wide and 400 m high. The data from these reflectors were converted into the line of sight of the satellites for comparison. In Fig. 4, the corrected time series of 24 of these reflectors are shown together with two ALOS-2 and two Sentinel-1 time series of points sampled in the area. The points chosen for the satellite data are approximately 50 to 200 m upslope from the reflectors, due to low coherence and loss of correlation in the area immediately around the reflectors. From December 2014 to June 2015 a very fast downslope displacement is recorded by all reflectors and such acceleration is not fully captured by the satellites. This may be caused by a combination of different factors: (i) the points of the time series do not correspond perfectly to the area reflectors, this likely leading to high variability in the local vertical to horizontal components ratio of the displacements, due to the high roughness of the terrain and high slope angle variability in the area of the 2013 failure,(ii) the total station reflectors are the representation of the displacement at a particular location, whilst the InSAR pixel in our processing is ~15 m, iii) there are large return periods in the first part of the ALOS-2 time series and gaps in the Sentinel-1 time series (see connection graph in Supplementary Fig. 2) over which the velocity is interpolated. However, from June 2015, the velocity shown by the reflectors decreases and the time series obtained with the satellite data reproduce this trend very well. An acceleration in June 2016 is shown by the reflectors and captured by ALOS-2 time series.

## Discussion and conclusions

A detailed chronology of the events occurring at the site since 2009 is not known to the authors, however, a few main points are established. Works at the site began in November 2008, large floods occurred in 2009, in 2013 a large failure occurred on the east-facing slope, centred around the dam axis and the deepest excavations. Since then, and at least up to October 2017 (but most likely for much longer), stabilisation measures have been under way, including long anchors and shear pile walls, as construction was halted. The first acceleration phase in sector B, thus in the area of the 2013 failure, in our time series is observed from 2009, followed by a sharper acceleration from 2010. Such accelerations could be a response of the slope to either high rainfall totals or to the undercutting of the toe caused by excavation works or, more likely, to a combination of these factors. In Supplementary Fig. 1, we show ALOS-1 and Sentinel-1 time series for point x in sector B (see Fig. 1 for location) in association with TMPA derived monthly rainfall estimates^27^. Here, it can be observed that the 2010 the acceleration is more significant than a seasonal increase in displacement rate associated with previous monsoon seasons and does not seem to be explained only by precipitation. Another sector of the slope, sector D, shows acceleration in a similar period in an area largely unaffected by works at the site. This possibly indicates that rainfall totals during the monsoon may be at least partly responsible for the increasing velocities here. However, whilst sector D remains largely unchanged through 2018 in terms of areal extent of displacements and displacements rates, these parameters both increase substantially for sector B. This seems to suggest that undercutting of the landslide toe in sector B has played a substantial role in triggering secondary slope failures at the toe of the large pre-existing landslide. The gap in our time series between February 2011 and September 2014 does not allow for speculation regarding the fact that the continued acceleration period in sector B between 2009 and 2011 is precursory of the 2013 failure. From September 2014 until mid-2017, ALOS-2 and Sentinel-1 show two main phases of acceleration. These two acceleration phases appear to be to some extent independent of rainfall rates, given that the first occurs during winter 2014 and that the second one sets the scene for a continuous trend well into the post-monsoon season. However, short periods of even faster rates likely related to monsoonal rainfall can be seen on top of the overall trends of ALOS-2 time series (between September and November 2014, July and August 2015 and July and September 2016). A key point of the analysis is that the period between end-2014 and mid-2017 seems to indicate that the stabilisation measures undertaken at the toe of sector B from 2013 onwards have not reduced the slope displacement rates and in fact, accelerations post 2013 were observed along with an increase in the extent of the displacements area (Fig. 3C). In 2016, a smaller secondary slope failure located above the main highway was triggered by excavation works for the highway. This event is also visible as an acceleration in the monitoring data of the right abutment (Fig. 4). The last period in our time series, from mid-2017 to end 2018 shows a large-scale decrease in the displacement velocities in comparison with the previous three years. The question could be raised as to whether the stabilisation activity at the site has contributed to decrease the displacement rates. However, there are indications that the monsoon seasons of 2017 and 2018 were characterised by lower rainfall totals than in 2016 (see Supplementary Fig. 1), which may have contributed to a reduction in the displacement rates associated with lower pore water pressures and, perhaps in part, to lower river erosion power. Despite the lower displacement rates observed through the last period, a new secondary failure occurred on January 22^nd^ 2019^17^, outside the period of our time series. This slope failure occurred in the lowermost section of the slope, where intensive excavation works for the right dam abutment had been made, but not supported intensively.

The overall area affected by active displacements is approximately 8 km^2^, when all sectors are considered, which is about 60% of the areal extent of the landslide covering the entire slope up to the head scarp. The well-defined, separate boundaries of the identified sectors may suggest that different, potentially shallow instabilities are present. However, there are a number of important observations that suggest that the entire valley flank may actually be a deep-seated mountain slope deformation, on which large, secondary rock or soil slides have developed. First of all, the head scarp and the morphology of the valley flank, including the bulging of the toe and the roughness of the terrain, are compatible with one large, deep seated instability. Moreover, intensely fractured and damaged rock was observed in outcrops scattered over an area estimated to be between 4 and 5 km^2^, encompassing sectors A and B. Though sector C was not accessed during the field visit, the displacements observed here would suggest a similar degree of internal deformation as observed in the southern portion of the slope. Sporadic observations in the northern half of the slope, indicated undisturbed residual soil, hence suggesting a lesser degree of internal deformation than the southern half. Indeed, the lower displacements and the smaller areas of sectors D and E indicate a lower degree of activity. Nonetheless, the northern part of the slope could also be part of the large instability, if a complex geometry of the basal shear plane could explain the differences in activity and surface observations.

Our data show that the area affected by significant downslope displacements in sector B reached a maximum extent of 1 km^2^. The 2013 failure has a surface area of about 0.11 km^2^ and a depth of up to 100 m as recorded by inclinometers in the slope, resulting in a volume of about 6 million m^3^. The area that is supported with stabilisation measures is about 0.02 km^2^ (the right abutment of the dam) and is about 50 times smaller than the area covered by sector B. An area of 1 km^2^ with a depth of 50 m (likely an underestimation) would imply volumes of about 50 million m^3^, thus at least an order of magnitude larger than the 2013 failure. Considering the scenario in which the entire valley flank is affected by deep-seated instability and in a state of critical stability, the volumes involved, potentially an order of magnitude larger than the scenario presented here for sector B, would dwarf the volumes of the 2013 failure. The depth of an instability affecting the entire valley flank would be, in fact, most likely associated with much greater depths than those hypothesised here and sector B would represent a shallower, secondary rock slide.

We observe that the sectors identified in this analysis are in some cases associated with secondary scarps or with deeply incised channels. For example, in sector A, the greatest displacements are quite evident under a scarp at 1360 m a.s.l., below the village. The more diffuse region of lower displacements extends upslope, and is bounded in the south by the flank of the presumed mountain slope instability and by a channel in the north. In sector D and sector E, two scarps mark the upper boundary of the maximum displacements. These scarps have a height of 7 and 16 m and the base at 1452 m a.s.l. and 1272 m a.s.l. respectively. In sector B, the first acceleration phase (February 2009 – February 2011) also corresponds to highest accelerations over a portion of the slope between 1350 and 1450 m a.s.l. This area shows a steep, linear profile just downslope of a flat bench and immediately upslope of the fresh scarps likely related to the 2013 failure. In the following acceleration phase, again velocities increasing downslope are observed in the same slope sector, between 1350 and 1450 m a.s.l. Moreover, in this period, accelerating displacement rates are observed also over a section upslope of this, between 1450 and 1600 m a.s.l. These observations could point at accelerations over increasingly high portions of the slope, in response to a retrogressive failure. Finally, the displacements observed in sector F, which are absent in the period of ALOS-1 and appear in the other two sensors, are related with intense modifications occurring at the ground surface, due to human activity. Google Earth images show modification of the ground surface between 2002 and 2013 and three large structures being built by 2014. The correlation of the location of the displacements with such geomorphological or surface features suggests that the sharp gradients observed are unrelated to artifacts, but real. Notably, however, the total areas affected by displacements of sectors A B, D and E appear neither to be bounded by specific morphological features nor correlated on the whole with slope gradients, which supports the hypothesis that the instability could have a significant depth.

Sati *et al*. (2017) state that no major landslide area can be observed on the right slope, with the exception of one location. This is found 1.3 to 1.5 km upstream of the dam axis and between 1260 and 1225 m a.s.l. and has an extent of 230 by 90 m. The location of this area seems to coincide with our sector D, though the area indicated by Sati *et al*. (2017) is much smaller. Another vulnerable zone that they recognised is 1.9 to 2.3 km upstream of the dam axis, thus possibly coinciding with our sector E. Even though their analysis indicates that no major stability problems around the reservoir rim should be encountered, our analyses points to a different direction. In particular, our study shows that 1) several areas show significant downslope displacements across the entire valley flank, 2) such displacements are already visible from 2007, thus before works at the dam site began, 3) an acceleration is visible in the lower parts of the slope, immediately upslope of the dam axis, from 2009 onwards, thus around the time excavation began, 3) since stabilisation efforts began in 2013 accelerations continued with increasing velocities observed in the lower parts of the slope well into 2017, this indicating the ineffectiveness of stabilisation efforts with respect to the large scale landslide body, 4) even disregarding the possibility that the whole valley flank is affected by a deep seated instability, the lowermost part of the slope, directly above the dam axis, is affected by significant displacements over an area that is much larger than the area affected by the 2013 landslide, 5) field observations of intensely fractured and deformed rock in different areas of the larger slope seem to point to the fact that the whole valley flank may indeed be affected by a large instability. Multitemporal DInSAR analyses revealed the presence of a previously unrecognised instability, moreover, despite the relatively small observation window, InSAR analyses alone would firstly allowed identification of the presence of the instability, through a non-targeted, whole frame processing and then the raising of concerns regarding the location of the dam in the initial phases of the project. This analysis shows the potential of DInSAR for 1) the assessment of the suitability of a site for a large hydropower project, 2) targeting of stabilisation measures and investigating their effectiveness, 3) understanding the spatial and temporal evolution of displacements and 4) long-term monitoring of the entire valley flank impinging on a hydropower dam. This type of case study and InSAR application for hydropower projects is still relatively poorly documented in the literature. The higher availability of InSAR data and processing platforms and the shorter revisit time of satellites, such as those of the Sentinel constellation, will make this tool increasingly accessible to larger groups of users and will allow better transparency at all stages of a project. This is particularly important in order to generate sufficiently widespread knowledge around these issues and to empower relevant monitoring authorities to guarantee the safety of communities and improve hazard understanding and land management. Given the considerable interests at stake in this sector and the fast growth of hydropower production, especially in developing countries, it is contended that studies of this type are important in making the technique and its specific application more widely understood.

## Data and methods

Data from ALOS-1, ALOS-2 and Sentinel-1 acquired over the period February 2007 – October 2018 were processed over the area. The data are summarised in Table 1. ALOS-1 and ALOS-2 are in L-band (wavelength ~23 cm), whilst Sentinel-1 is in C-band (wavelength ~5.6 cm). The longer wavelength of the ALOS-1 and 2 sensors has the advantage of allowing better coherence in longer baseline interferograms, due to a lower sensitivity to vegetation. On the contrary, the shorter wavelength of Sentinel-1 is more affected by vegetation changes between successive acquisitions, hence it is more difficult to obtain interferograms with high correlation at longer (>2 months) temporal baselines. By virtue of the wavelength difference, though, ALOS-1 and 2 are less sensitive to slow displacement and are better suited for the detection of faster displacements, whilst Sentinel-1 can capture slower movements too. A more in-depth description of the advantages and disadvantages of different wavelengths and of the benefits of multi-sensors analyses can be found in Dini *et al*. (2019). Initially, a number of interferograms were generated, across the entire available frames using the software SARScape developed by Sarmap. The interferograms in the wrapped phase were analysed individually and displacements over the study area of this paper were first identified^28^. Successively, velocity and cumulative displacements maps were generated with the Small Baseline Subset (SBAS) method^29^. After the first, large-scale, velocity and displacement maps generation and in order to mitigate against unwrapping errors, a cut of the SAR images was made around the study area, covering roughly 15 km^2^ directly encompassing the slope and the immediate surroundings. In this way, problems related to coherence islands disconnected by incoherent areas, were avoided. The unwrapped interferograms obtained with this second step of the analysis were analysed individually, to inspect for large unwrapping errors before the time series generation. The absence of significant unwrapping errors allowed the retention of all interferograms for ALOS-1 and ALOS-2. A number of interferograms of Sentinel-1 data were discarded for either lack of coherence or unwrapping errors. Images that lost all connections and were thus discarded are shown in Supplementary Fig. 2 as small diamonds. All connection graphs are shown in Supplementary Fig. 2. Maximum temporal baselines were set at 750, 365 and 48 days for ALOS-1, ALOS-2 and Sentinel-1 respectively, whilst maximum perpendicular baselines are 3500, 280 and 190 m respectively. The high control over perpendicular baselines of ALOS-2 allowed for the generation of SBAS time series even with an exiguous number of images. Temporal baselines were kept at 48 days for Sentinel-1 due to the highest sensitivity to changes in land cover given by the shorter wavelength of C-band. Disconnected blocks of images were used to construct the Sentinel-1 time series. This was necessary due to several images being discarded, as mentioned above, given the low coherence of the interferograms that they generated. The model fits a linear velocity, interpolating between blocks of images, where no connections exist. The topographic model used for interferometric processing is the 5 m horizontal resolution ALOS World3d digital surface model. Reference points were chosen on areas deemed stable, at the crest of the slope and on the ridges to the south and north, bounding the instability and immediately outside of the presumed landslide boundaries, avoiding the valley bottom. Areas of cumulative displacements > 20 mm in the period of observation of each sensor were mapped and time series of points within such areas were analysed. 3 trend changes were identified in the time series of ALOS-1 and Sentinel-1. Linear velocities before and after such changes were estimated and the velocities analysed along a profile across the instability impinging on the dam.

With the purpose of validation only, we summarise and compare with the InSAR results some *in-situ* data. A field visit in October 2017 allowed for the identification of some important morphological features. Moreover, *in situ* data were obtained from the Punatsangchhu Hydroelectric Project Authority (PHPA). The data includes time series of displacements occurring at 39 reflectors placed within and upslope of the 2013 instability and measured by a total station installed on the left side of the dam. The data of 24 of these reflectors were converted with trigonometrical calculation into the line of sight (LOS) of the satellites and then compared to the InSAR time series. Depending on the acquisition geometry, the downwards, eastwards and northwards components of the displacement vector are:

1 $${\rm{d}}=\,\sin \,\vartheta $$

2 $${\rm{e}}=\,\cos \,\vartheta \,\cos \,{\rm{\phi }}$$

3 $${\rm{n}}=\,\cos \,\vartheta \,\sin \,{\rm{\phi }}$$

where ϑ is the complementary angle to the incidence angle and φ is the angle between the LOS and the east. The components of the vectors for correction of the *in-situ* data are shown in Table 2. After the three components are corrected, the new vector projected on the LOS is obtained through the sum of the three components.

**Table 2:** Corrections used for projection of *in-situ* data on satellites LOS.

	ALOS-1	ALOS-2	Sentinel
ϑ	90°−39°	90°−31°	90°−39°
φ	16°	16°	12°
d	0.77	0.85	0.77
e	0.131	0.44	0.131
n	0.615	0.53	0.615

## Supplementary information

Supplementary figures.
