# Supplementary material for: The Punatsangchhu-I dam landslide illuminated by InSAR multitemporal analyses
Source: Sci Rep. 2020 May 19;10:8304. doi: 10.1038/s41598-020-65192-w (PMC7237427; doi:10.1038/s41598-020-65192-w)
Supplement: Supplementary file 1 — Supplementary figures. [file 41598_2020_65192_MOESM1_ESM.pdf]

SUPPLEMENTARY FIGURES

The Punatsangchhu-I dam landslide illuminated by InSAR multitemporal analyses

Benedetta Dini[1*], Andrea Manconi[1], Simon Loew[1], Jamyang Chophel[2]

[1] Department of Earth Sciences, ETH Zurich, Switzerland

[2] PHPA-I, Wangduephodrang, Bhutan

[*]b.dini@uea.ac.uk

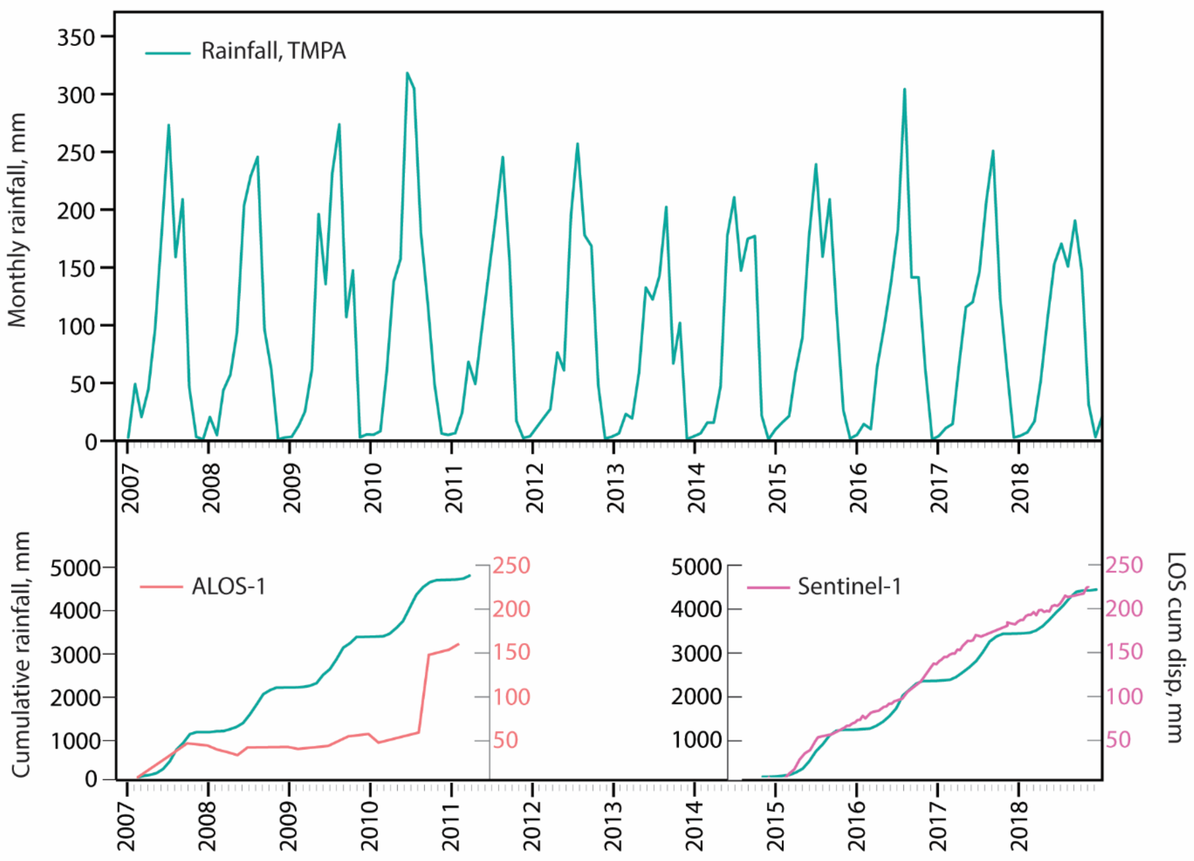

Supplementary fig. 1. Top: estimated rainfall from TMPA multi-satellite precipitation analysis for 27.375° N, 89.875° E. The monthly rainfall is estimated based on the average hourly rates (TRMM, 2011). Bottom: time series of LOS displacements from ALOS-1 (left) and Sentinel-1 (right) for point x in sector B (Fig. 2). InSAR time series are superposed to cumulative rainfall calculated from TMPA data.

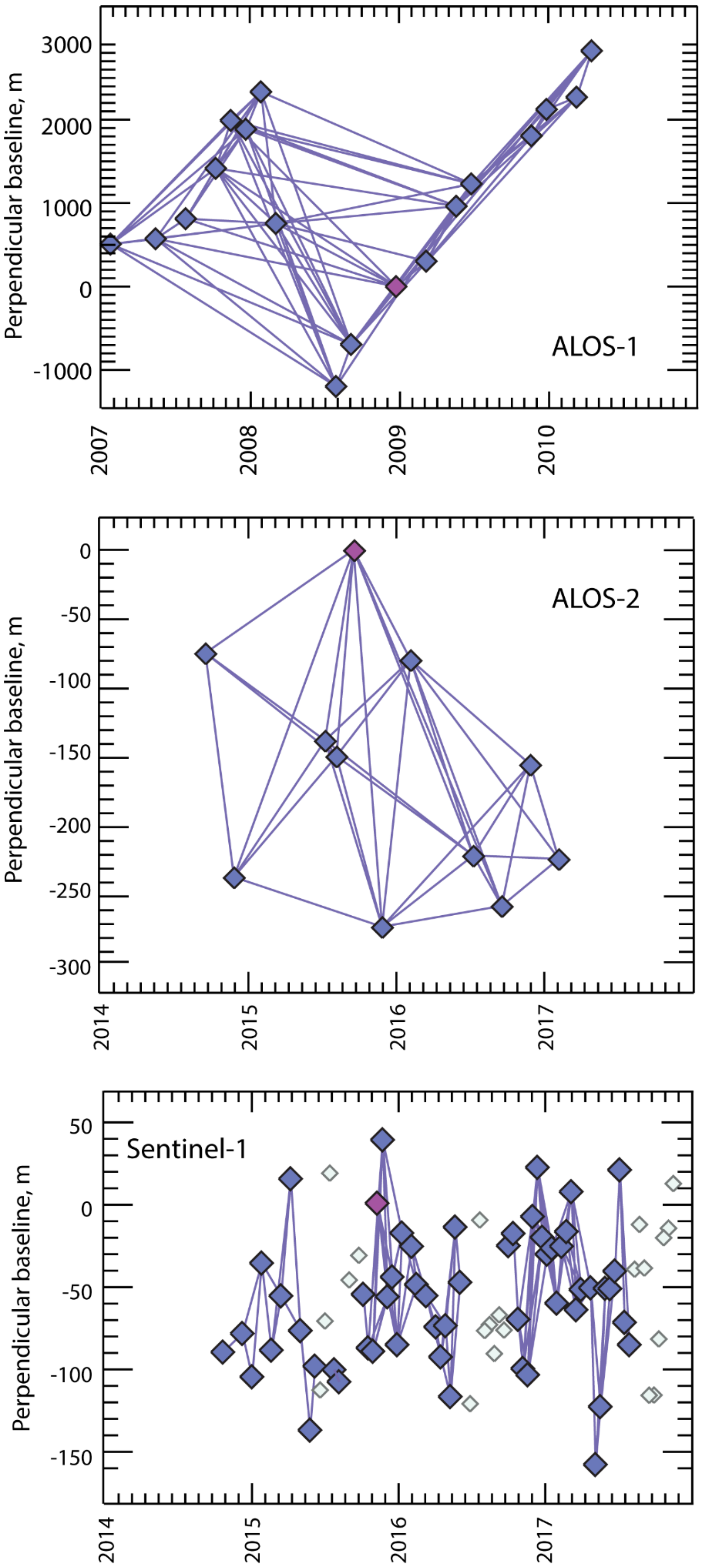

Supplementary fig. 2. Connection graphs of the SBAS processing for the three sensors. Small diamonds in the bottom panel represent images that were discarded for the time series generation. ALOS-1 and ALOS-2 SAR imagery obtained from JAXA (Dataset ©JAXA/METI ALOS PALSAR L1.0 2007, 2008, 2009, 2010, 2011, 2014, 2015, 2016, accessed through https://auig2.jaxa.jp/openam/UI/Login, 2016 and 2017). Copernicus Sentinel data [2014, 2015, 2016, 2017, 2018] accessed through the ESA Copernicus open access hub https://scihub.copernicus.eu/dhus/#/home, 2017, 2018.

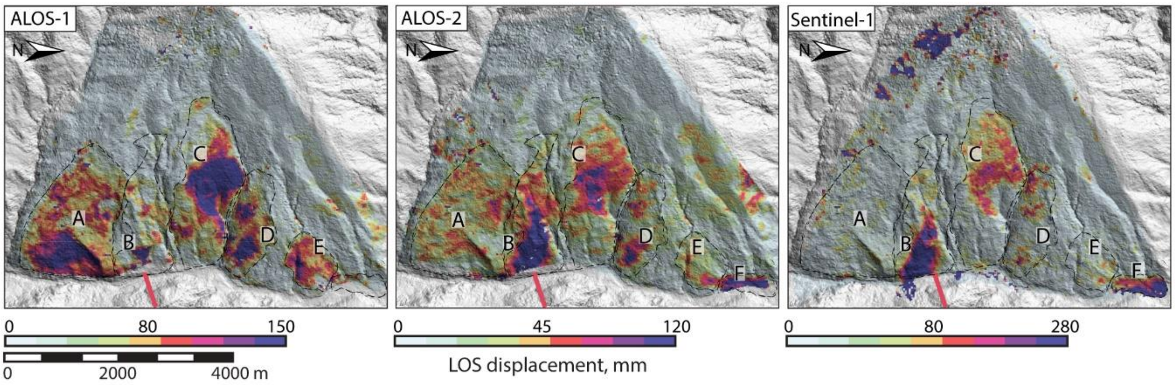

Supplementary fig. 3. LOS displacements projected onto the maximum slope gradient. The correction applied to the LOS is based on the C-coefficient model of Notti et al. (2011) [1], which is a model aimed at evaluating the percentage of real detectable movement. This is based on the assumption that the movement occurs along the line of maximum slope gradient and that the velocity projected along such gradient is the ratio between the LOS velocity and a coefficient (C-coefficient). Here we see that the downslope velocity is higher than the LOS in many parts of the slope. For example, in the observation period of Sentinel-1, the true downslope cumulative displacement in sector B could be as high as 200% higher than the LOS cumulative displacement, reaching 900 mm in places (scales are saturated as per Fig. 3 in the main text). Black dashed lines represent the maximum extent of the sectors described in section 2.1.

ALOS-1 and ALOS-2 SAR imagery obtained from JAXA (Dataset ©JAXA/METI ALOS PALSAR L1.0 2007, 2008, 2009, 2010, 2011, 2014, 2015, 2016, accessed through https://auig2.jaxa.jp/openam/UI/Login, 2016 and 2017). Copernicus Sentinel data [2014, 2015, 2016, 2017, 2018] accessed through the ESA Copernicus open access hub https://scihub.copernicus.eu/dhus/#/home, 2017, 2018. InSAR processing done with the software SARScape from Sarmap.

1. Notti, D., Meisina, C., Zucca, F. & Colombo, A. Models to predict Persistent Scatterers data distribution and their capacity to register movement along the slope. *Fringe 2011 Workshop, 19–23 September 2011, ESA/ESRIN, Frascati* (2011).
